# Gene Regulatory Evolution During Speciation in a Songbird

**DOI:** 10.1534/g3.116.027946

**Published:** 2016-03-10

**Authors:** John H. Davidson, Christopher N. Balakrishnan

**Affiliations:** Department of Biology, East Carolina University, Greenville, North Carolina 27858

**Keywords:** Dobzhansky-Muller, genome, inviability, reproductive isolation, sterility

## Abstract

Over the last decade, tremendous progress has been made toward a comparative understanding of gene regulatory evolution. However, we know little about how gene regulation evolves in birds, and how divergent genomes interact in their hybrids. Because of the unique features of birds – female heterogamety, a highly conserved karyotype, and the slow evolution of reproductive incompatibilities – an understanding of regulatory evolution in birds is critical to a comprehensive understanding of regulatory evolution and its implications for speciation. Using a novel complement of analyses of replicated RNA-seq libraries, we demonstrate abundant divergence in brain gene expression between zebra finch (*Taeniopygia guttata*) subspecies. By comparing parental populations and their F1 hybrids, we also show that gene misexpression is relatively rare among brain-expressed transcripts in male birds. If this pattern is consistent across tissues and sexes, it may partially explain the slow buildup of postzygotic reproductive isolation observed in birds relative to other taxa. Although we expected that the action of genetic drift on the island-dwelling zebra finch subspecies would be manifested in a higher rate of *trans* regulatory divergence, we found that most divergence was in *cis* regulation, following a pattern commonly observed in other taxa. Thus, our study highlights both unique and shared features of avian regulatory evolution.

The study of gene expression in diverging species and their hybrids provides insights into the mechanisms of regulatory network evolution, adaptation, and the origins of postzygotic reproductive isolation. Of particular interest to the process of speciation is gene misexpression, where expression in hybrids falls outside the range of variation observed in both parental populations (*i.e.*, over- or underdominance). Misexpression in hybrids may reflect Dobzhansky-Muller type incompatibilities and thus, can highlight the genetic changes underlying such incompatibilities (Michalak and Noor 2004). Over the last decade, the comparative scope of gene regulatory evolution studies has expanded to include diverse study systems [*e.g.*, *Drosophila*: Landry *et al.* 2005; *Xenopus*: Malone and Michalak 2008; whitefish (*Coregonus spp*.): Renaut *et al.* 2009; yeast: Emerson *et al.* 2010; Busby *et al.* 2011; Schaefke *et al.* 2013]. However, to date no such study has been conducted in birds.

Birds display a number of traits that make them a particularly interesting target for studies of speciation genomics. First, they display female heterogamety, where females are ZW and males are ZZ for their respective sex chromosomes. This feature allows for independent testing of sex chromosome-related features of speciation. For example, faster molecular evolution on the avian Z chromosome has been shown (Mank *et al.* 2010; Nam *et al.* 2010; Balakrishnan *et al.* 2013; Wright *et al.* 2015), following a pattern observed in many other taxa with heterogametic males (reviewed in Meisel and Connallon 2013). Therefore, in terms of gene expression, we may expect to see faster expression evolution in Z-linked genes and a tendency for Z-linked genes to be misexpressed in hybrids. Second, the evolution of reproductive isolation is protracted in birds relative to other taxa (Prager and Wilson 1975; Fitzpatrick 2004; Price 2008). Astoundingly, fully fertile hybrids have been documented from bird species that have diverged for up to 10 million years (Tubaro and Lijtmaer 2002; Lijtmaer *et al.* 2003; Price 2008; Arrieta *et al.* 2013). In many other taxa, studies of gene expression have pointed to frequent misexpression in F1 hybrids (Landry *et al.* 2005; McManus *et al.* 2010; Malone and Michalak 2008; Renaut *et al.* 2009; Bell *et al.* 2013; Coolon *et al.* 2014). If gene misexpression in hybrids is reflective of the buildup of postzygotic reproductive incompatibilities, then we may expect to see a reduced frequency of misexpression in bird species relative to other taxa of similar age.

The advent of RNA-seq technology has added a new dimension to the study of regulatory evolution, as it is now possible to estimate the relative expression of alternative alleles across all expressed polymorphisms (McManus *et al.* 2010; Bell *et al.* 2013). Because F1 hybrids share the same *trans* regulatory environment, allelic imbalance (allele-specific expression) in F1 hybrids allows the further categorization of regulatory divergence into the contributions of *cis* and *trans* regulatory evolution and the interaction of the two (Wittkopp *et al.* 2004). Most interspecific comparisons to date have found *cis* divergence to be more common (Landry *et al.* 2005; Tirosh *et al.* 2009; Goncalves *et al.* 2012; Graze *et al.* 2009). However, others have found a larger than expected contribution of *trans* divergence (McManus *et al.* 2010; Coolon *et al.* 2014). Based on comparisons between *Drosophila melanogaster* and *D. sechellia*, McManus *et al.* (2010) hypothesized that demographic differences (increased drift) may drive this higher frequency of *trans* divergence. They posited that *trans* acting differences tend to generate intraspecific expression polymorphisms (Lemos *et al.* 2008; Wittkopp *et al.* 2008; Emerson *et al.* 2010), which might in turn be fixed by drift (Coolon *et al.* 2014).

Although there is extensive information about fertility and viability loss in hybrid birds (Tubaro and Lijtmaer 2002; Lijtmaer *et al.* 2003; Price 2008; Arrieta *et al.* 2013), to date there have been no studies of regulatory divergence in bird species and their hybrids. While zebra finches (*Taeniopygia guttata*) are an established model system for the neurobiology of song learning (Clayton *et al.* 2009), they also have great potential for mechanistic studies of speciation. In this study, we examine regulatory divergence in two zebra finch subspecies, both of which are available in captivity and thus are readily amenable to experimental study. *Taeniopygia g. castanotis* and *T. g. guttata* inhabit mainland Australia and the Lesser Sunda islands of Southeast Asia, respectively. The Australian subspecies is broadly distributed across inland Australia whereas the Lesser Sundan subspecies (hereafter “Timor”) is found on the islands east of Wallace’s Line, a well-known biogeographic barrier (Wallace 1863; Huxley 1868). The subspecies appear to have diverged approximately one million years ago (Balakrishnan and Edwards 2009) when zebra finches colonized the Lesser Sunda islands from Australia (Mayr 1944). The two subspecies are reciprocally monophyletic for mtDNA alleles (Newhouse and Balakrishnan 2015), but not for any nuclear markers surveyed to date (Balakrishnan and Edwards 2009). Patterns of genetic variability suggest the colonization of the islands involved a substantial population bottleneck that is reflected in much reduced genetic diversity among island birds (Balakrishnan and Edwards 2009). Here, we broadly describe patterns of expression divergence between zebra finch subspecies and, in doing so, test whether genetic drift resulting from a historical bottleneck has impacted patterns of regulatory evolution in zebra finches.

## Materials and Methods

### RNA extraction, library preparation, and sequencing

Birds were housed in captivity at the Institute for Genomic Biology at the University of Illinois at Urbana-Champaign. Three male birds were sampled from each of three populations: Australian (*T. guttata castanotis)*, Timor (*T. guttata* guttata), and hybrid finches. All of the hybrid birds studied were the result of crosses between female Australian zebra finches and Timor males. This crossing directionality was chosen because female Australian zebra finches breed more readily in captivity than female Timor finches. In order to control for environmental influences on gene expression, each individual bird was placed in an acoustic isolation chamber the night before they were to be euthanized. To avoid pharmacological influences on gene expression, birds were then euthanized by decapitation. Tissues were dissected and then snap-frozen on dry ice. All animal protocols were approved by the University of Illinois IACUC. All procedures subsequent to dissections were carried out at East Carolina University.

Whole brain tissue was homogenized in Tri-Reagent (Molecular Research Company) for RNA purification and total RNA was extracted following the manufacturer’s instructions. Total RNA was then DNase treated (Qiagen) to remove any genomic DNA contamination and the resulting RNA was further purified using RNeasy columns (Qiagen). Purified total RNA was assessed for quality using an Agilent Bioanalyzer. Library preparation and sequencing were done at the University of Illinois Roy J. Carver Biotechnology Center. Library preparation used Illumina TruSeq RNA Sample Prep Kit and the manufacturer’s protocols. RNA sequencing was performed in a single lane of an Illumina HiSeq 2000 using a TruSeq SBS sequencing kit version 3, producing single end 100 bp reads, which were analyzed with Casava 1.8.2. Reads were adapter and quality-trimmed using Trim Galore! (Kreuger 2015), a wrapper script that uses cutadapt (Marcel 2011) to trim reads.

### Read mapping, expression measurement, and differential expression testing

We expected that reads from Australian zebra finches would map to the reference genome (v3.2.74) at a higher rate than Timor zebra finches because the genome was derived from the Australian subspecies. We observed such biases in preliminary analyses using bwa aln (Li *et al.* 2009a) and tophat2 (Kim *et al.* 2013; Table 1). We observed little bias, however, when we mapped reads to the genome using bwa mem (Li 2013) under default settings (Table 1). Therefore, we used this read aligner for subsequent analyses. Despite the apparently consistent mapping of reads at the whole genome scale, mapping bias at specific loci that are divergent in sequence could still preclude accurate expression measurements. To avoid this, we masked sites in the genome with fixed differences between the three individuals of each subspecies (see Supplemental Material, File S3). To accomplish this, we identified polymorphisms in the dataset using samtools mpileup (Li *et al.* 2009b) and called SNPs using bcftools (Li *et al.* 2009b). Fixed differences were then identified using SNPSift (Cingolani *et al.* 2012), filtering the VCF (variant call format) file generated by bcftools for sites that were homozygous for the reference allele in the three Australian birds and homozygous for the alternative allele in the Timor birds. These sites were then masked in the reference genome using bedtools (Quinlan and Hall 2010). Following masking, we remapped reads to the masked genome again using bwa mem. The proportion of mapped reads dropped by about 1% after masking fixed differences (Table 1) but we used the masked mapping to avoid any potential bias in downstream analyses.

**Table 1:** Total number of reads after quality trimming and proportion mapped to the reference genome before and after masking polymorphic sites

	Trimmed Reads	tophat2 Initial	tophat2 Masked	bwa mem Initial	bwa mem Masked
Australian					
Library 1	31,726,619	0.684	0.472	0.9	0.885
Library 2	33,049,620	0.669	0.455	0.876	0.871
Library 3	32,844,330	0.669	0.455	0.874	0.869
Average		0.674	0.461	0.883	0.875
Timor					
Library 1	33,115,938	0.643	0.464	0.883	0.879
Library 2	34,328,721	0.645	0.466	0.883	0.879
Library 3	33,467,969	0.621	0.443	0.864	0.86
Average		0.636	0.458	0.877	0.873

We quantified gene expression relative to Ensembl-annotated gene models (Ensembl v73). For each gene, we counted the number of overlapping reads using ht-seq (Anders *et al.* 2014). We then used DE-Seq2 (Anders and Huber 2010; Love *et al.* 2014) to normalize read counts per library and to test for differential expression. We conducted four pairwise tests to categorize genes as differentially expressed among species, but also to categorize inheritance as dominant/recessive, additive, or over/underdominant. Together, we consider the latter two categories as being “misexpressed.” The pairwise tests were: Australian (n = 3) *vs.* Timor (n = 3), hybrids (n = 3) *vs.* parentals (n = 6), hybrids (n = 3) *vs.* Australian (n = 3), and hybrids (n = 3) *vs.* Timor (n = 3). Inheritance was considered additive if expression in hybrids was intermediate to the two parentals but not significantly different from either parental subspecies. If hybrid expression was intermediate, but expression in hybrids was significantly different from one parent but not the other, inheritance was considered dominant. Genes were considered misexpressed when hybrids were significantly different from both parental populations. Patterns other than these were considered ambiguous.

### Allele-specific expression and mechanisms of regulatory divergence

We used the allelic depth (DP) field in the VCF file generated by bcftools to estimate the coverage of alternative alleles in each library. We restricted the allelic expression analysis to the sites identified previously as having fixed differences between subspecies. In order to examine patterns of allelic expression, we generated a data matrix containing counts for each site in each parental sample, and counts of each allele in hybrid samples. Thus, the final data matrix contained 12 columns, one for each of the six parental samples, and two for each of the three hybrid samples (one for each allele). The site-level matrix of count data was normalized for read-depth in DE-Seq2, and differential expression tests were then used to identify sites showing significant regulatory divergence. For each site, we conducted a test of differential allellic expression in the hybrids and for differential expression between the parental subspecies.

Evidence of biased allelic expression in F1 hybrids is a result of *cis* regulatory divergence (Wittkopp *et al.* 2004). *Trans* divergence is identified by comparing the ratio of expression in the parents and the ratio of expression of the alleles in hybrids (Wittkopp *et al.* 2004). To identify genes with significant *trans* regulatory divergence, we constructed a linear model in DE-Seq2 with two main terms: “type,” which denotes whether reads were allelic counts in the parental species (note that each parental species only expresses one allele as these are fixed differences) or allelic counts in hybrids, which express both alleles. The second term describes the “condition,” whether counts are of allele A or B. The model also then included an interaction between condition and type,

design(transTest) <- formula(∼ type + cond + type:cond)

resTransTest <-results(transTest, name=”typeE.condB”)

where typeE specifies parental expression (as opposed to allelic) and condB specifies allele B count. The “type” term in the model controls for differences in the counts between parental measurements and allelic measurements. This results function tests the null hypothesis that the ratio of allele A and B in the parental subspecies is equal to the allelic ratio of A to B in the hybrids. All tests were considered significant if the FDR-adjusted *P* value was less than 0.1.

Sites were categorized as *cis*-only if there was a significant expression difference between subspecies and there was allele-specific expression, but there was no evidence of *trans* divergence (Table 2). *Trans*-only divergence was inferred if there was a difference between the subspecies, there was no allele-specific expression in hybrids, but there was *trans* divergence. If there was divergence in *cis* and *trans*, these could be further parsed into *cis* + *trans* and *cis* × *trans* based on whether parental divergence and allelic imbalance were in the same, or opposite direction, respectively. Compensatory evolution, a subcategory of *cis* × trans interactions, was inferred if there was no difference in expression between subspecies but there was evidence of divergence in both *cis* and *trans*. Sites that showed no parental, *cis*, or *trans* divergence were considered conserved, and sites that did not fit any of these categories were considered ambiguous. Sites were functionally annotated using SNPeff (Cingolani *et al.* 2012), which uses the reference genome and annotation to determine where polymorphic sites are located relative to gene models. Although our primary analysis looked at regulatory divergence at the SNP level, we also examined gene level patterns using annotations imported from

**Table 2:** Overview of classification scheme for categorizing patterns of regulatory divergence

Mode	Parental Divergence	ASE in Hybrids	TransTest
*cis*-only	Yes	Yes	No
*trans*-only	Yes	No	Yes
*cis* × *trans*, *cis* + *trans*	Yes	Yes	Yes
Compensatory	No	Yes	No
Conserved	No	No	No

“Yes” or “no” refers to a significant statistical test as defined in the *Materials and Methods*. ASE, allele-specific expression.

SNPeff.

### Data availability

The authors state that all data necessary for confirming the conclusions presented in the article are represented fully within the article.

## Results

### Differential expression between subspecies

Nine RNA-seq libraries, derived from RNA extracted from the brains of three Australian, three Timor, and three F1 hybrid zebra finches, yielded over 30 million reads per sample (Table 1). All data have been deposited in the NCBI short read archive under project SRP071222. Using bwa mem (Li 2013), we were able to map over 85% of our reads to a version of the zebra finch genome that had been masked of SNPs fixed for alternative alleles in our sample of Australian and Timor finches (see *Materials and Methods*). Across all nine libraries, we detected 16,689 out of 18,618 (89.6%) Ensembl-annotated genes with at least one read in one library.

After filtering for variance outliers under default settings in DE-Seq2 (Anders and Huber 2010), 13,904 genes were tested for differential expression. Of these, 913 genes (6.6%) were differentially expressed between Australian and Timor zebra finches (*P* < 0.05; see File S1). All *P* values from DE-Seq2, Gene Ontology (GO), and KEGG pathway analyses were adjusted for multiple testing (Benjamini and Hochberg 1995). Of the differentially expressed genes, 51.5% were expressed at a higher level in Australian finches and the remaining 48.5% were expressed more highly in Timor zebra finches. Thus, the distribution of fold changes across all genes was centered around zero with no tendency of genes to be up-regulated in one population *vs.* the other. Among the differentially expressed genes, those with roles in the oxidation-reduction process (GO:0055114, 44 genes, Fisher’s Exact Test, *P* = 0.0085) and oxidoreductase activity (GO:0016491, 36 genes, *P* = 0.014) were significantly enriched (see File S2). In both of these GO categories, just over 50% (52.3% and 52.7%, respectively) of the transcripts were more highly expressed in Australian Zebra Finches. Genes with annotated roles in protein binding, sequence-specific DNA binding, and transcription factor activity were underrepresented (*P* < 0.05), suggesting relatively conserved expression of genes in these categories. No KEGG pathways were significantly enriched or underrepresented after correcting for multiple testing. However, among the differentially expressed genes, those with roles in oxidative phosphorylation (KEGG: gga00190), were slightly enriched (observed = 10, expected = 4, *P* = 0.17). Eight out of these 10 oxidative-phosphorylation-related genes were more highly expressed in Timor finches relative to Australian zebra finches (binomial test, *P* = 0.04).

### Expression divergence on the sex chromosome

We tested for elevated regulatory divergence on genes of the Z chromosome between subspecies by comparing the variance in fold-changes across the Z, to that of genes on chromosome 4, the chromosome most similar in size to the Z. Constraining evolutionary rate comparisons to comparisons between similarly sized chromosomes is important, because evolutionary rate is associated with recombination rate, which is in turn associated with chromosome size in birds (Kunstner *et al.* 2010). If Z chromosome genes were diverging more rapidly in terms of expression, we would expect a larger variance in fold change. However, we found no significant difference in variance among chromosomes (F = 1.03, *P* = 0.72). We also found no enrichment of genes on the Z chromosome among those that were differentially expressed between subspecies. Whereas Z-linked genes comprise 4.6% of the detected genes in our dataset (793/13,904), 3.6% (33) Z-linked genes were differentially expressed. This difference was not statistically significant (χ^2^ = 2.02, *P* = 0.15). Thus, Z-linked genes are not significantly over- or underrepresented among the differentially expressed genes.

### Inheritance of gene expression

**Figure 1:**
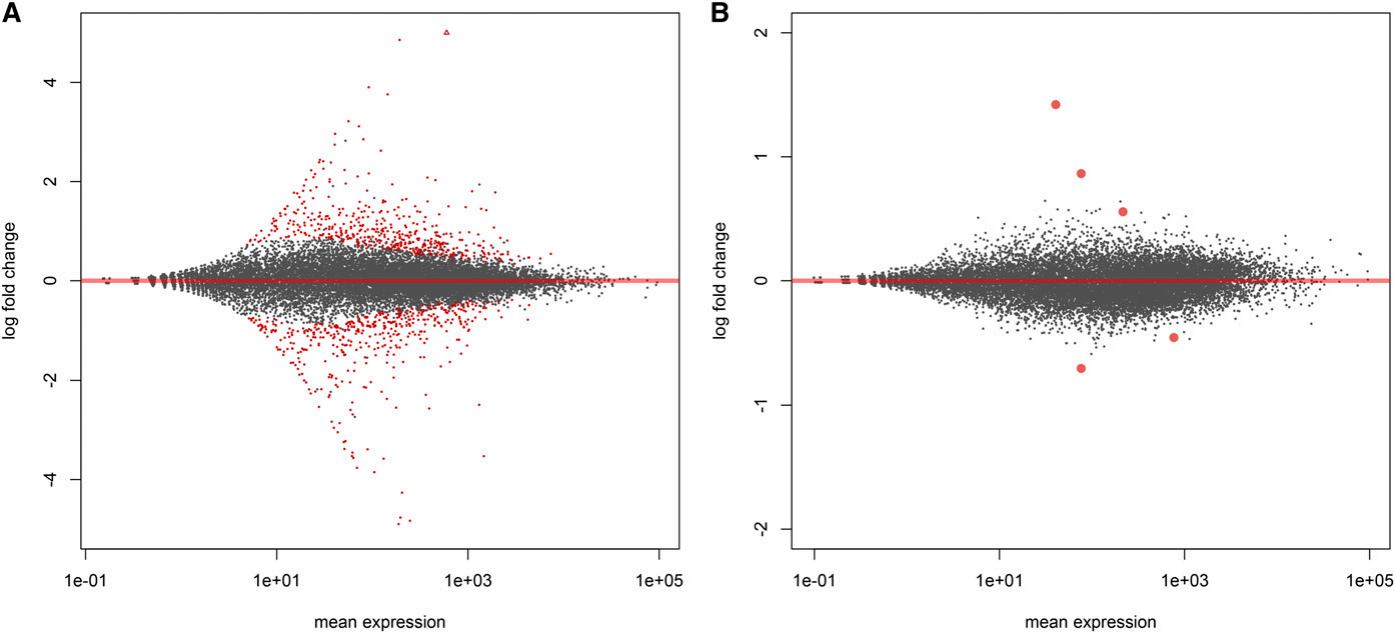
MA plot (expression level *vs.* log fold change) of differential expression for two contrasts. (A) Australian *vs.* Timor zebra finches and (B) Parental subspecies *vs.* their hybrids. Points in red are significant at *P* < 0.05 (adjusted for multiple testing). Larger point size in panel B is simply to increase visibility.

**Figure 2:**
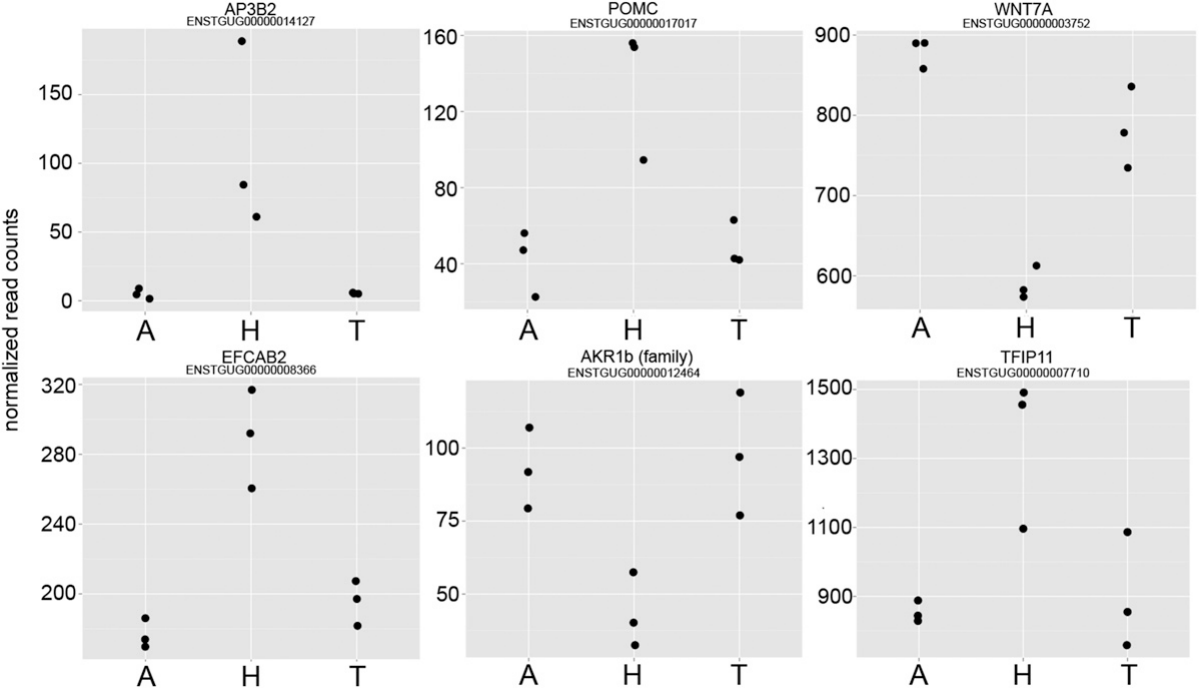
Six misexpressed genes in hybrid zebra finches. Statistics are based on differential expression comparison of the two zebra finch subspecies (n = 6) *vs.* their hybrids (n = 3) (Figure 1B). Five of these genes are significant at adjusted *P* < 0.05 and the sixth (TFIP11) is significant at adjusted *P* < 0.1 A, Australian; H, Hybrid; T, Timor.

We also classified the mode of inheritance of expression profiles in hybrid birds relative to the parental subspecies. We successfully classified inheritance for 847 genes. By contrasting expression in both subspecies (n = 6 samples) *vs.* their hybrids (n = 3), we found only five genes (0.5%) with significant evidence of misexpression in hybrids (*P* < 0.05; Figure 1B and Figure 2): AP3B2, POMC, WNT7A, EFCAB2, and AKR1b (gene family member). At a less stringent significance threshold of *P <* 0.10, only one additional gene, TFIP11, can be classified as misexpressed (Figure 2). Thus, in contrast to many previous studies in non-avian taxa, misexpression was relatively rare. Instead, the vast majority of genes showed an additive inheritance pattern. Among the genes differentially expressed between subspecies, 631 in total (74.5%) showed an additive pattern. Another 211 genes showed evidence of dominance. Among the genes that showed dominance, the Timor zebra finch expression pattern was dominant over that of the Australian zebra finch in 169 genes, and only 42 showed the reverse pattern. Thus, there was a significant tendency for the Timor expression pattern to be dominant (χ^2^ = 70.6, *P* < 0.0001).

### Mode of regulatory divergence

Biased allelic expression in F1 hybrids reflects *cis* regulatory divergence between parents, since alleles inherited from each parent are exposed to the same *trans* regulatory environment (Wittkopp *et al.* 2004). Unlike previous RNA-seq studies of regulatory divergence (*e.g.*, McManus *et al.* 2010; Bell *et al.* 2013; Coolon *et al.* 2014), we used an experimental design with biological replicates. Therefore, we were able to use statistical software tailored for RNA-seq, thus incorporating observed variance profiles within and among genes to test for both allelic bias in hybrids and *trans* divergence. *Trans* divergence is identified in genes that show differences in allelic expression ratio in hybrids as compared to that observed between parental populations (Wittkopp *et al.* 2004, see *Materials and Methods*). We assessed patterns of allelic expression in 23,838 SNPs whose genotype was ascertained for all nine samples. This set of SNPs included only sites for which our sample of the two subspecies was fixed for alternative alleles, allowing unambiguous determination of ancestry in the F1 hybrids. Of the SNPs we tested for allele-specific expression, only 6634 (28%) mapped within annotated genes (including exons, annotated untranslated regions (UTRs), and introns). The majority of SNPs, 12,129 (50.1%) in total, mapped outside of gene models but within 5 kb downstream of known genes, possibly representing unannotated UTR regions. Because the annotation of the zebra finch genome is incomplete, gene associations of these and other noncoding SNPs are uncertain. Thus, we first conducted our analysis at the level of individual sites rather than genes (Bell *et al.* 2013), recognizing that some SNPs will be nonindependent because they are associated with the same gene.

**Figure 3:**
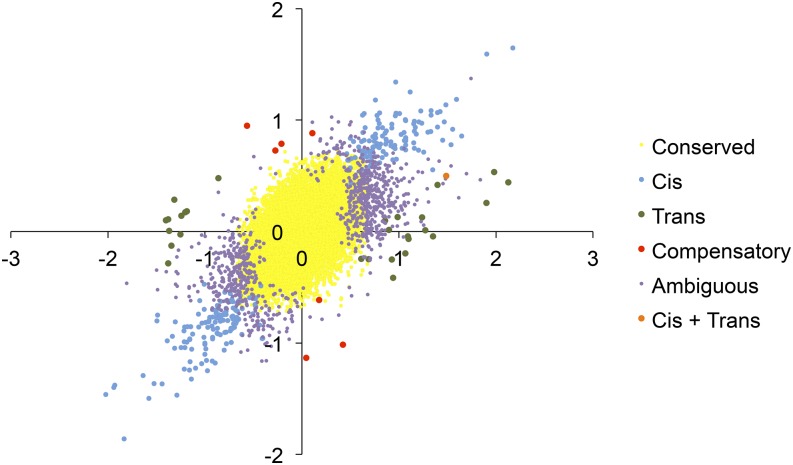
Categorization of regulatory divergence modes based on patterns of allele-specific expression in hybrids and subspecific divergence. Most loci showed conserved expression (yellow). However, among those that show significant evidence of evolution, changes in *cis* regulation were most common (light blue). Less frequently observed categories are depicted with larger symbols to increase visibility.

If genetic drift has led to an accumulation of deleterious alleles in Timor zebra finches, one pattern we might expect to see is a tendency toward higher expression of Australian alleles (*e.g.*, Bachtrog 2006; Tuttle *et al.* 2016). In general, however, the two alternative alleles were expressed equally in hybrid birds (22,658/23,838 SNPs, 95%). Of the remaining sites, we found significant evidence of biased allelic expression, and thus *cis* divergence between parents, in 253 SNPs (1% of all sites, Figure 3). Two hundred and twenty-five of the 253 SNPs were putatively associated with 155 annotated genes (in the UTR, intron, exon, or within 5 kb up or downstream) and the remaining 28 SNPs were intergenic. Even among the sites where we observed biased expression in hybrids, the average log2 fold change was zero. Thus, there was no bias in terms of which allele (Timor or Australian-derived) was more highly expressed.

We combined information from allelic bias in F1 hybrids with information on expression differences between parental subspecies and a test for *trans* divergence to further categorize regulatory divergence into subcategories (Figure 3 and Table 2). Another 53 sites showed significant *trans* divergence. Of these, 28 sites putatively representing 17 genes showed *trans*-only divergence. Seven sites showed evidence of compensatory evolution, where there was *cis* and *trans* regulatory divergence, but no net divergence in overall expression between subspecies. Only one site showed significant divergence in expression between subspecies, significant *cis* divergence, and significant *trans* divergence. This represents a lone case of *cis* and *trans* regulatory divergence acting together to cause expression divergence between subspecies. Eight hundred and ninety-one sites revealed an ambiguous pattern and could not clearly be categorized in their mode of divergence.

Although, as mentioned above, gene models for zebra finches are uncertain, we assigned all of these SNPs to 6983 gene regions to conduct these same analyses at the gene level. We assigned SNPs within 5 kb of a particular gene to that gene, as UTRs in particular are poorly annotated. Analysis at the gene level showed a similar pattern to SNP level analyses, with predominant divergence in *cis* regulation. Fifteen genes showed *trans* regulatory divergence while 122 showed *cis* divergence. One gene showed both *cis* and *trans* divergence, and another three showed evidence of compensatory changes.

## Discussion

In this study we have broadly characterized the regulatory divergence of brain-expressed transcripts in two zebra finch subspecies that have been geographically isolated for around one million years (Balakrishnan and Edwards 2009). We find evidence of abundant expression divergence between the two populations, with over 900 brain-expressed genes showing differential expression. GO analyses revealed that, among those differentially expressed genes, those involved in oxidation-reduction and metabolic processes were significantly enriched. The divergence in genes associated with metabolism, including a mild enrichment of oxidative phosphorylation-related genes, could be related to ecological adaptation to different habitats in inland Australia *vs.* the Lesser Sunda islands. Alternatively, it is possible that differences in expression are the result of short-term adaptation to captivity in the Australian subspecies, which has a longer domestication history (∼150 years, Zann 1996). The tendency for genes in the oxidative-phosphorylation KEGG pathway to be more highly expressed in Timor zebra finches (8 out of 10 differentially expressed genes) suggests the possibility that these changes may be a result of adaptation rather than drift.

Unlike many previous studies (Landry *et al.* 2005; McManus *et al.* 2010; Malone and Michalak 2008; Renaut *et al.* 2009; Bell *et al.* 2013; Coolon *et al.* 2014), we found that misexpression was rare among the genes we measured in hybrid zebra finches. Even though we found subspecies differences in the expression of genes related to cellular energetics, we did not find any evidence of misexpression of mitochondrial or other oxidative phosphorylation-related genes in hybrids. Mito-nuclear interactions are known to contribute to genomic incompatibilities in certain taxa (*e.g.*, Burton *et al.* 2006; Ellison and Burton 2006; Ellison *et al.* 2008) and have recently been suggested to be particularly likely candidates as “speciation genes” (Burton and Barreto 2012; Hill 2015). In zebra finches, we know that mtDNA alleles are differentiated between the two species (Newhouse and Balakrishnan 2015), yet we don’t find evidence of resultant misregulation of mitochondrial genes.

A number of factors may contribute to the low levels of misexpression in zebra finch F1 hybrids. Although the two zebra finch subspecies are relatively divergent, they are young relative to some species pairs previously tested for misexpression (*e.g.*, *D. simulans* × *D. melanogaster*, 2.5 million years, Landry *et al.* 2005; *Sacchromyces cerevisiae* × *S. paradoxus*, 5 million years, Tirosh *et al.* 2009). On the other hand, even crosses between relatively young mouse subspecies (< 0.5 million years) exhibit hybrid sterility and misexpression (Mack *et al.* 2016). Given that there is no evidence of reproductive incompatibility between the zebra finch subspecies and that postzygotic isolation takes a relatively long time to evolve in birds (Prager and Wilson 1975), our results suggest that misexpression may accumulate after the origin of reproductive incompatibilities or may directly contribute to the origin of incompatibilities.

It is important to note that we examined only brain tissue in this study. If patterns of expression in the brain are relatively conserved, that could contribute to the reduced levels of misexpression observed here. Unlike many studies of *Drosophila*, which examine whole organism expression profiles, Graze *et al.* (2009) examined gene expression in dissected heads of *D. melanogaster* × *D. simulans* hybrids. They too found limited evidence of misregulation (∼30 genes at FDR < 0.2) suggesting that misregulation might be less common in the brain. In the present study, however, we did observe divergence in expression of over 900 brain-expressed genes, suggesting abundant regulatory divergence between subspecies without resultant misexpression in F1 hybrids. Examination of F2 or backcrossed individuals remains an important goal for the future, as second generation crosses allow recombination among parental genomes, potentially exposing deleterious allelic combinations. For example, studies of wild whitefish populations showed relatively little misexpression in F1 hybrids (9% of genes) but abundant misexpression in backcrosses (54% of genes, Renaut *et al.* 2009). The importance of second generation hybrids is particularly true for incompatibilities derived from mito-nuclear interactions where such crosses can pair a mitochondrial genome from one genetic background with the nuclear genetic background of the other.

The most interesting possible implication of our findings is if the lack of misexpression in zebra finch hybrids is related to the long-known pattern of slow postzygotic reproductive isolation evolution in birds (Prager and Wilson 1975). Thorough testing of this hypothesis, however, will require examination of hybrids of both sexes, additional tissues, and species pairs. A number of hypotheses for the slow buildup of incompatibilities in birds have been proposed. Fitzpatrick (2004) favored a hypothesis of slower regulatory evolution in birds than in mammals. Our results do not support this hypothesis as we observed substantial amounts regulatory divergence between subspecies, yet little evidence of misregulation in hybrids. Another hypothesis is that differences in dosage compensation and sex chromosome systems are responsible (Fitzpatrick 2004). The idea is that in mammals, X inactivation in females causes deleterious recessive mutations on the X chromosome to be exposed in both sexes, whereas in birds, males express their diploid Z chromosome genotype. In this study we tested only males, thus it is possible that an examination of females would expose more widespread misregulation of sex-linked genes and their interaction partners. A novel hypothesis is that the slow rate of evolution of postzygotic incompatibility is due to the stability of the avian karyotype (*e.g.*, Griffin *et al.* 2007). If changes in genomic architecture (*e.g.*, interchromosomal rearrangements) contribute to regulatory divergence and hence, to genetic incompatibilities, it may be that it simply takes longer for such changes to accrue in birds.

The small number of misregulated genes does not necessarily imply that misregulation is unimportant in this system. One gene that was clearly misexpressed is proopiomelanocortin (POMC, Figure 2). POMC is notable as a gene with multifaceted roles in pigmentation and social behavior. Pigmentation patterns clearly differ among zebra finch subspecies (Clayton 1990) and aspects of social behavior may vary as well. It will be of interest to determine whether regulatory changes in POMC or any other misexpressed gene contributes to any phenotypic differences in the zebra finch subspecies, and whether the misexpression that does occur causes aberrant phenotypes in hybrid offspring.

Studies of sex chromosome evolution have revealed that, like the mammalian X chromosome, the avian Z chromosome is evolving rapidly relative to autosomes. This pattern has been attributed primarily to genetic drift (Mank *et al.* 2010), and is further modulated by variation in the strength of sexual selection (Wright *et al.* 2015). However, we found no unusual patterns of expression divergence for Z-linked genes. This finding underscores the results of a recent study that found that the fast Z effect on gene expression in birds was limited to expression patterns from female gonadal tissue (Dean *et al.* 2015). At the nucleotide level, the Z chromosome has been demonstrated to be evolving relatively quickly in estrildid finches (Balakrishnan *et al.* 2013), the group to which zebra finches belong. These changes must not be influencing gene expression in the male brain, or compensatory substitutions may be mitigating the consequences of deleterious changes. We were, however, only able to detect a handful of compensatory changes, and none of these were on the Z chromosome.

The Timor zebra finch subspecies has undergone a severe bottleneck in colonizing the Lesser Sunda islands, as evidenced by dramatically reduced neutral genetic variation (Balakrishnan and Edwards 2009). That pattern, as well as patterns of morphological trait evolution, led to the conclusion that a founder effect likely played a role in zebra finch speciation (Balakrishnan and Edwards 2009). Under strong genetic drift, we expected to see relatively abundant *trans* regulatory divergence if many deleterious alleles were fixed in the island populations (McManus *et al.* 2010). Such a pattern was observed in comparisons of *D. melanogaster* and *D. sechellia*, the latter of which is also an island form (McManus *et al.* 2010). In zebra finches, however, as in a number of recent studies (Landry *et al.* 2005; Tirosh *et al.* 2009; Goncalves *et al.* 2012; Mack *et al.* 2016), we find that most of the regulatory divergence was *cis* acting. We also predicted that under strong drift, deleterious mutations would accumulate and impair normal levels of gene expression. Such patterns have been observed in situations where recombination is suppressed (*e.g.*, neo sex chromosomes: Bachtrog 2006; “supergenes”: Tuttle *et al.* 2016). In this case, Timor zebra finch alleles would show a tendency to be underexpressed relative to the Australian allele in heterozygous hybrids. Again though, we do not see this pattern. Taken together, we do not find compelling evidence of bottleneck-induced drift influencing patterns of gene expression.

Finally, on a technical note, we presented a simple method by which both differential expression and allelic specific expression (and thus, the contributions of *cis* and *trans* divergence) can be assessed in a consistent statistical framework using replicated experiments and statistics tailored for RNA-seq data. Previous RNA-seq-based studies of regulatory divergence have estimated allelic bias using binomial tests, which do not take into account sample variance or the expected and observed pattern of variance in RNA-seq data. Here, we have used a single software package, DE-Seq2, to test for divergence in gene expression, allelic expression, and the interaction of the two, or *trans* divergence. Specifically, we used an interaction term in a general linear model to test for *trans* divergence. One caveat, which appears to be common to various tests of regulatory divergence and requires further examination, is that *cis* and *trans* tests may differ in statistical power (*e.g.*, Suvorov *et al.* 2013). Divergence (spread) along the *cis* axis tends to be greater (Figure 3, see also Mack *et al.* 2016), even in cases of abundant *trans* divergence (*e.g.*, McManus *et al.* 2010). Furthermore, due to the incorporation of biological variation, a large number of sites and genes are left with ambiguity in their mode of inheritance. Nevertheless, the incorporation of biological variation using DE-Seq2 (or similar approaches) is essential. Replicated experimental designs, paired with phylogenetic studies of regulatory divergence (*e.g.*, Coolon *et al.* 2014) will continue the progress toward a broad, comparative understanding of regulatory evolution.

## Supplementary Material

Supplemental Material
