# Supplementary material for: Gene Regulatory Evolution During Speciation in a Songbird
Source: G3 (Bethesda). 2016 Mar 10;6(5):1357–64. doi: 10.1534/g3.116.027946 (PMC4856086; doi:10.1534/g3.116.027946)
Supplement: Supplemental Material [file supp_6_5_1357__index.html]

Gene Regulatory Evolution During Speciation in a Songbird — Supplemental Material 

# Gene Regulatory Evolution During Speciation in a Songbird

## Supplemental Material for Davidson and Balakrishnan, 2016

**Files in this Data Supplement:**

- File S1 - Differential expression statistics from the comparison of Australian and Timor Zebra Finches in DE-Seq2. (.xlsx, 2 MB)
- File S2 - Gene Ontology (GO) and Kyoto Encylopedia of Genes and Genomes (KEGG) enrichment analysis of differentially expression genes between Australian and Timor Zebra Finches. (.xlsx, 139 KB)
- File S3 - Sample code used to generate a masked reference genome. This procedure identifies fixed differences between subspecies and masks those sites in the genome. This new masked reference genome is used in downstream analyses to avoid potential mapping bias. (.txt, 6 KB)
